# A Significant Association between Type 1 Diabetes and *Helicobacter pylori* Infection: A Meta-Analysis Study

**DOI:** 10.3390/medicina60010119

**Published:** 2024-01-09

**Authors:** Wei-Kian Chua, Yi-Kai Hong, Shu-Wei Hu, Hueng-Chuen Fan, Wei-Hsin Ting

**Affiliations:** 1 Division of Pediatric Endocrinology, Department of Pediatrics, Tungs’ Taichung MetroHarbor Hospital, Taichung 43503, Taiwan; chuaweikian@gmail.com; 2 Department of Dermatology, National Cheng Kung University Hospital, College of Medicine, National Cheng Kung University, Tainan 70101, Taiwan; jack810325@gmail.com; 3 International Center for Wound Repair and Regeneration (iWRR), National Cheng Kung University, Tainan 70101, Taiwan; 4 Department of Dermatology, Feinberg School of Medicine, Northwestern University, Chicago, IL 60611, USA; 5 Division of Pediatric Gastroenterology, Department of Pediatrics, Tungs’ Taichung MetroHarbor Hospital, Taichung 43503, Taiwan; 6 Ph.D. Program in Translational Medicine, National Chung Hsing University, Taichung 40227, Taiwan; 7 Rong Hsing Research Center for Translational Medicine, National Chung Hsing University, Taichung 40227, Taiwan; 8 Division of Pediatric Neurology, Department of Pediatrics, Tungs’ Taichung MetroHarbor Hospital, Taichung 43503, Taiwan; 9 Department of Rehabilitation, Jenteh Junior College of Medicine, Nursing and Management, Miaoli 35664, Taiwan; 10 Department of Pediatric Endocrinology, MacKay Children’s Hospital, Taipei 10449, Taiwan; 11 Department of Medicine, MacKay Medical College, New Taipei 25245, Taiwan

**Keywords:** *Helicobacter pylori*, Type 1 diabetes mellitus, HbA1c, child, children, young patients, pediatrics, adolescents, meta-analysis

## Abstract

*Background and Objectives*: Type 1 diabetes mellitus (T1DM) is a chronic and serious condition that is characterized by inadequate pancreatic-β-cells’ insulin production. The connection between T1DM and *Helicobacter pylori* infection remains uncertain. This study aimed to conduct a systematic meta-analysis to examine the association between *H. pylori* infection, hemoglobin A1c levels, and the development of T1DM. *Materials and Methods*: The initial search identified 451 articles on the association between *H. pylori* infection and T1DM. Among them, 12 articles had 2797 participants who met the inclusion criteria for an advanced meta-analysis. *Results*: A significant association was observed between *H. pylori* infection and T1DM (OR 1.77, 95% CI 1.47–2.12, *p* < 0.0001), with heterogeneity: Tau^2^ = 0.47; Chi^2^ = 57.07, df = 11 (*p* < 0.0001); I^2^ = 81%. Subgroup analysis showed that *H. pylori* infection was significantly associated with a longer duration of T1DM and higher hemoglobin A1c levels (*p* < 0.001 for both) but not with age at T1DM diagnosis (*p* = 0.306). *Conclusions:* These findings contribute to the understanding of the association between *H. pylori* infection and T1DM and highlight the potential role of *H. pylori* in influencing the duration and glycemic control of diabetes. Therefore, pediatric patients who have longstanding T1DM and poor glycemic control should be screened for *H. pylori* infection.

## 1. Introduction

Type 1 diabetes mellitus (T1DM) is a chronic and notably severe medical condition that is recognized for its defining feature of an inadequate production of insulin by the pancreatic β-cells. Typically, this condition has been conventionally associated with its predominant occurrence in the pediatric and young adult demographic. Nonetheless, it is imperative to note that T1DM can potentially manifest across a broad spectrum of ages, dispelling the notion that it is solely confined to the younger population [1]. Notably, a substantial majority (85% to 90%) of individuals with T1DM have autoantibodies that specifically target key proteins, including insulin, glutamic acid decarboxylase 65, insulinoma-associated autoantigen 2, zinc transporter 8, and tyrosine phosphatase IA-2β. This underscores the complex interplay of immune responses and genetic predisposition, which plays a pivotal role in the pathogenesis of T1DM [2,3]. Autoimmune destruction of β cells is the critical precursor of the potential onset of T1DM. Therefore, immediate and precise diagnosis is important to manage this condition effectively. Timely diagnosis not only facilitates the implementation of appropriate medical interventions but can also prevent the complications of T1DM. By recognizing and addressing the condition at an early stage, healthcare practitioners can significantly enhance the overall quality of life of individuals with T1DM.

*Helicobacter pylori* is a prevalent Gram-negative bacterium that has a notable pathogenic profile and can spread widely to a substantial percentage (i.e., >50%) of the worldwide population. *H. pylori* infection has a substantial ubiquity in developing nations, with prevalence rates of approximately 85% to 95%. In contrast, in developed countries, this prevalence assumes a range of 30% to 50%, underscoring a significant disparity influenced by socioeconomic and environmental factors [4,5]. An intriguing facet of this epidemiological landscape is the noteworthy downward trajectory observed in the prevalence of *H. pylori* infection across European countries since the dawn of the twenty-first century. This trend hints at evolving factors such as changes in lifestyle, improved hygiene practices, and advances in healthcare that have contributed to a decline in the incidence of this infection within these geographical areas. Conversely, Asian countries exhibit a relatively stable prevalence of *H. pylori* infection over the same time frame, suggesting that the factors that influence the persistence of this infection vary across different regions. The intricate interplay between genetics, cultural practices, dietary habits, and healthcare infrastructure potentially contributes to this stability in the prevalence of *H. pylori* in Asian populations [5,6]. *H. pylori* infection exerts a wide-ranging influence on human health, exerting its effects not only on gastric systems but also on various extra-gastric systems. *H. pylori* infection has a spectrum of complications that extend beyond the stomach and carry consequences that encompass both well-established gastric issues and an array of less commonly recognized extra-gastric disorders. For gastric complications, *H. pylori* infection orchestrates a cascade of pathological events that encompass gastritis, peptic ulcer disease, functional dyspepsia, gastroesophageal reflux disease, and the formidable specter of gastric cancer. These interlinked conditions collectively constitute a spectrum of disturbances that highlight the intricate interplay between *H. pylori* and the gastric environment. However, the impact of *H. pylori* extends far beyond the stomach and is intricately connected with several extra-gastric complications. The repercussions of *H. pylori* infection transcend the anatomical confines of the stomach and may include cardiopulmonary diseases, such as coronary artery disease and asthma; hematologic disorders, including iron deficiency anemia and immune thrombocytopenic purpura; and neurologic maladies like ischemic stroke, Parkinson’s disease, Alzheimer’s disease, Guillain–Barré syndrome, and migraines. Moreover, the influence of *H. pylori* delves into dermatologic ailments, exemplified by its link to chronic spontaneous urticaria, as well as metabolic disorders that encompass metabolic syndrome and insulin resistance. Evidently, the diverse and intricate effects of *H. pylori* infection underscore its pivotal role in human health and emphasize its capability to affect multiple bodily systems. This intricate interplay and the potential to manifest in multifaceted ways further exemplify the need for a comprehensive understanding and management of *H. pylori* infection [7,8,9,10].

Numerous comprehensive studies have consistently demonstrated a robust and noteworthy correlation between the presence of *H. pylori* infection, a bacterial infection primarily affecting the stomach lining, and the notable elevation of the level of hemoglobin A1c (HbA1c) [11,12,13,14], which is a pivotal biomarker of long-term blood sugar control and reflects the average blood sugar level over a span of several months. This correlation had been particularly pronounced in a subset of individuals with type 2 diabetes mellitus (T2DM), which is a metabolic disorder that is characterized by high blood sugar levels secondary to insulin resistance. The intriguing findings of these previous studies indicated that *H. pylori* infection might exert a tangible influence on glycemic control, as evidenced by the increased HbA1c levels. Moreover, previous studies that were conducted across diverse geographical regions and populations have consistently shown a clear trend of a greater likelihood of *H. pylori* infection in individuals with T1DM than in their nondiabetic counterparts. This observation led to the necessity of elucidating the intricate interplay between *H. pylori* and the autoimmune processes that underlie the development of T1DM. The increased serological prevalence rate of *H. pylori* infection in patients with T1DM compels the need to investigate the potentially shared mechanisms or immunological conditions between these seemingly disparate conditions. Scientists have meticulously studied the possible immunomodulatory effects of *H. pylori* infection in the context of T1DM and sought to ascertain the contribution of this infection to the complex autoimmune cascade that leads to β cell destruction [15]. Hence, in light of the existing knowledge gaps and the imperative to comprehensively grasp the intricate relationships at play, the primary objective of the present research endeavor was to meticulously undertake a systematic meta-analysis. This endeavor, undertaken with methodological rigor and precision, sought to discern and quantify the overarching effect size that characterizes the intricate interplay among several pivotal factors, including *H. pylori* infection, HbA1c levels, and the eventual onset of T1DM in pediatric patients.

Numerous studies have shown a positive association between *H. pylori* infection and diabetes [16] and that, among the most common chronic diseases in children and adolescents, *H. pylori* infection was a potential risk factor for diabetes and T1DM [17,18]. However, we recognize the lack of pediatric data and aimed to conduct this meta-analysis to determine the association of *H. pylori* infection with HbA1c levels and the development of T1DM in pediatric patients. 

## 2. Materials and Methods

### 2.1. Data Collection

This meta-analysis was performed and reported in compliance with the Preferred Reporting Items for Systematic Reviews and Meta-Analyses (PRISMA) Statement [19,20]. All original articles were searched for on international databases, including NCBI (PubMed), ISI Web of Science, EMBASE, and Cochrane Library, without language limitations. The search strategy was conducted using the PICOS tool, which uses data on population (children), intervention (diabetes), outcomes (*H. pylori* infection), and study type (case–control study). The complete list of searched keywords was Diabetes, Diabetes Mellitus (type 1), Insulin Dependent, IDDM, Insulin Sensitivity, Helicobacter pylori, campylobacter pylori, H Pylori, Child, Children, Young patients, Pediatric, and Adolescents. The search was performed using the Medical Subject Headings and free keywords. The keywords selected for the search were (Children or Child or Young patients or Pediatric or Adolescents) and [Diabetes or T1DM or Diabetes Mellitus (type 1) or Insulin Dependent or IDDM or Insulin Sensitivity] and (*Helicobacter pylori* or *Campylobacter pylori* or *H. pylori*).

The studies were identified independently by two investigators, and disagreements were resolved by consensus. In the case of duplicate studies, only the most recent and informative publications were included in the analysis. Articles meeting the following inclusion criteria were evaluated: (1) case–control studies, (2) those with patients aged <20 years, (3) those that used *H. pylori* as an exposure variable. Case reports, reviews, meta-analyses, cross-sectional studies, and cohort studies were excluded from the analysis. Two investigators independently extracted the following data from eligible studies: year, nation, study design, sample size, proportion of sex, age, control group, odds ratio, type of diabetes, test method for *H. pylori*, and the control variable. 

### 2.2. Statistical Analysis

The meta-analysis was performed in accordance with the PRISMA guidelines [19]. Odds ratios (ORs) and standard errors were used to describe the results. When required, the OR with 95% confidence interval (CI) was calculated using MedCalc (https://www.medcalc.org/calc/odds_ratio.php/) (Version 22.017; accessed on 14 October 2023). Differences in the mean and standard deviation values were used for subgroup analysis. The random-effect model was used to combine the estimated effects [21]. Statistical heterogeneity was assessed using the I^2^ test, with I^2^ representing the proportion of the total outcome variability that was attributable to the variability among the studies [22].

All statistical analyses were performed using the Comprehensive Meta-Analysis (CMA) software 4.0 (Biostat Inc., Englewood, NJ, USA), and a *p*-value of <0.05 was considered statistically significant.

## 3. Results

### 3.1. Characteristics and Methodologies of the Included Studies

**Figure 1:**
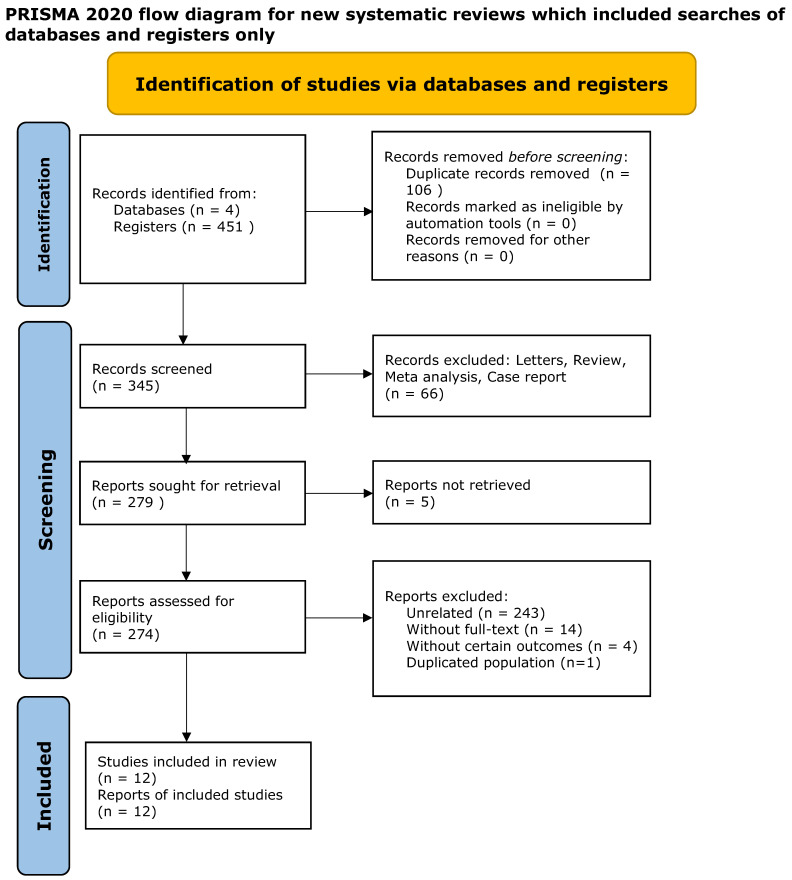
A PRISMA flowchart showing the study selection and inclusion process.

The PRISMA flowchart (Figure 1) shows the study selection and inclusion process. The initial phase of the research endeavor encompassed a comprehensive search that yielded a substantial corpus of 451 articles exploring a potential association between *H. pylori* infection and T1DM. We excluded 439 articles using a systematic filtering process. This exclusion covered a range of factors, including 106 duplicate articles, 267 pieces with outcomes not directly pertinent to the investigation, as well as 66 articles categorized as reviews, letters, meta-analysis studies, and case reports. Following this rigorous culling, a meticulously curated set of 12 articles were selected and emerged as prime candidates for the study, as illustrated in Figure 1 [23,24,25,26,27,28,29,30,31,32,33,34].

**Figure 2:**
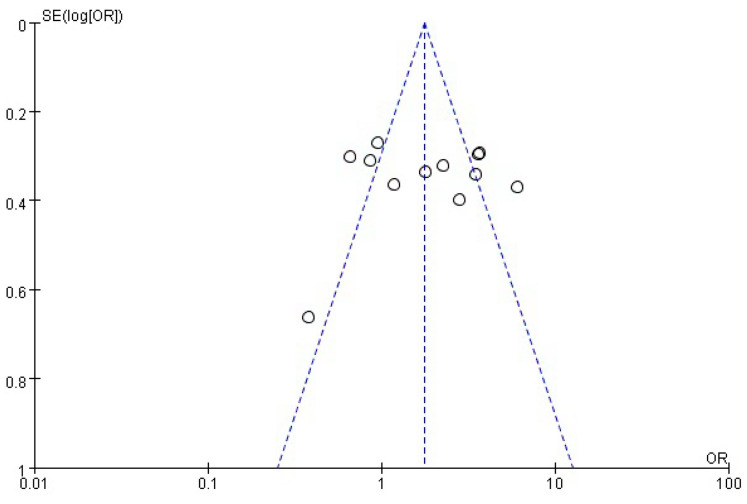
Funnel plot analysis of *H. pylori* infection and Type 1 diabetes mellitus.

Notably, the diversity of this pool was marked by one study, which included pediatric participants without specific age details; the remaining studies included patients aged 10–20 years. A comprehensive delineation of the salient features of these chosen studies is provided in Table 1. Overall, this advanced meta-analysis synthesized data from a noteworthy cohort of 2797 participants with T1DM. Delving deeper, the distribution between cases and controls came to 1159 and 1638, respectively, encapsulating a substantial breadth of relevant data for a comprehensive investigation into the potential relationship between *H. pylori* infection and T1DM. In six studies, *H. pylori* infection was diagnosed using the enzyme-linked immunosorbent assay. The remaining studies used other methods, such as the ^13^C urea breath test, RIBASIA, rapid urease test, enzyme immunoassay (EIA), and stool antigen test. To ensure the integrity of the findings and to ascertain the potential impact of publication bias, a methodical assessment was undertaken. This endeavor involved the construction of a funnel plot, a graphical representation that capitalizes on the logarithm and logarithm standard error of the odds ratio (OR) values attributed to *H. pylori* infection. The primary purpose of this construction was to visualize the distribution of studies in relation to their precision and effect size. Upon meticulous examination of the funnel plot, a distinct symmetrical pattern emerged, indicating a seemingly uniform spread of studies across the range of effect sizes. This observation, while suggestive, necessitated a more quantitative and robust analysis to corroborate the absence of publication bias. In this pursuit, the Begg’s rank correlation method was harnessed, which is a statistical tool designed to probe for potential correlations between study size and effect size. The outcomes of this analytical venture showcased a statistically non-significant result (Pr > |z| = 0.656 > 0.05), thereby indicating that the distribution and effect size of the studies were not influenced by publication bias. This result conferred a high degree of confidence in the impartiality and credibility of the research landscape and effectively ruled out systematic distortion of the analyzed data (Figure 2).

### 3.2. Meta-Analysis Results

**Figure 3:**
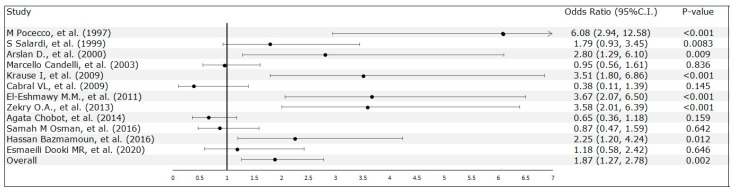
Correlation analysis of *H. pylori* infection and Type 1 diabetes mellitus [23,24,25,26,27,28,29,30,31,32,33,34].

An in-depth analysis of the quantitative breakdown showed that the prevalence of *H. pylori* infection was higher in the diabetes cohort (45.87%, n = 561) than in healthy individuals (24.27%, n = 413). This result highlighted a marked discrepancy in the prevalence of *H. pylori* infection between the two cohorts. Moreover, *H. pylori* infection was significantly associated with T1DM (OR 1.77, 95% CI 1.47–2.12, *p* < 0.0001), with heterogeneity: Tau^2^ = 0.47; Chi^2^ = 57.07, df = 11 (*p* < 0.0001); I^2^ = 81% (Figure 3).

Data, including age at diagnosis of T1DM, HbA1c levels, and duration of diabetes, were comprehensively collected from patients with diabetes for an advanced subgroup analysis. The participants within this study were thoughtfully categorized into distinct groups: the H. pylori-positive (HP+) group and the H. pylori-negative (HP−) group. This discerning division of participants enabled a focused and granular analysis, allowing for the disentangling of the complex interplay between H. pylori infection and the specific subsets characterized by distinct variables such as age at diagnosis, HbA1c levels, and duration of diabetes. By delving into this dichotomy of HP+ and HP− groups, the research team endeavored to unveil the association between H. pylori infection and these respective subgroups (Table 2).

**Figure 4:**
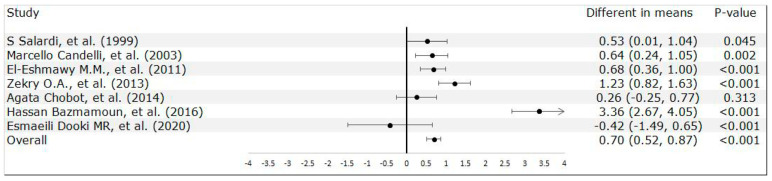
Correlation analysis of *H. pylori* infection and diabetes duration [24,26,29,30,31,33,34].

**Figure 5:**
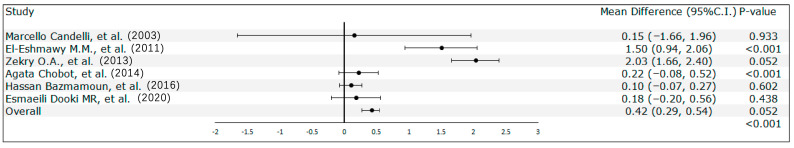
Correlation analysis of *H. pylori* infection and hemoglobin A1c levels [26,29,30,31,33,34].

Interestingly, this meticulous analysis found a nonsignificant association (*p* = 0.306) between *H. pylori* infection and age at T1DM diagnosis in this pediatric population. This absence of a significant association implied an inherent complexity in the relationship between *H. pylori* infection and T1DM and encouraged a deeper exploration of the factors that contributed to this result (heterogeneity: Chi^2^ = 18.21, df = 6 (*p* = 0.006); I^2^ = 67%) (Supplementary Figure S1). On the other hand, *H. pylori* infection was significantly associated with a prolonged duration of diabetes (*p* < 0.001; heterogeneity: Tau^2^ = 22.97; Chi^2^ = 554.16, df = 6, *p* < 0.00001; I^2^ = 99%) (Figure 4) and high HbA1c levels (*p* < 0.001; heterogeneity: Tau^2^ = 0.63; Chi^2^ = 105.34, df = 5, *p* < 0.00001; I^2^ = 95%) (Figure 5). The multifaceted relationships of *H. pylori* infection with age at T1DM diagnosis, diabetes duration, and HbA1c levels suggested that presence of and the need to further explore the intricate interactions and underlying mechanisms.

### 3.3. Sensitivity Analysis Results

Sensitivity analysis was performed using the statistical software Comprehensive Meta-Analysis (CMA) 4.0. Among the seven studies included, the OR was 2.30 (95% CI 1.79–2.96, *p* < 0.001; heterogeneity: Chi^2^ = 14.61, df = 6, *p* = 0.11; I^2^ = 40%) (Supplementary Figure S2A). The difference in the mean age had an OR of 0.13 (95% CI −0.12 to 0.38; heterogeneity: Tau^2^ = 0.19; Chi^2^ = 6.21, df = 4, *p* = 0.18; I^2^ = 36%) (Supplementary Figure S2B). The difference in the mean duration of diabetes had an OR of 0.70 (95% CI 0.52–0.87; heterogeneity: Tau^2^ = 0.07; Chi^2^ = 7.74, df = 6, *p* = 0.10; I^2^ = 48%) (Supplementary Figure S2C). The difference in the mean HbA1c level had an OR of 0.42 (95% CI 0.29–0.54; heterogeneity: Chi^2^ = 22.10, df = 5, *p* = 0.0002; I^2^ = 82%) (Supplementary Figure S2D). 

## 4. Discussion

In this meta-analysis, *H. pylori* infection was found to have a positive association with elevated HbA1c levels and duration of T1DM in pediatric patients. These results were in accordance with those of previous worldwide studies. The seroprevalence of *H. pylori* was reported to be significantly higher in patients with T1DM than in healthy control individuals [29,30]. In one study on patients with diabetes, the prevalence of *H. pylori* infection was relatively high in those who were aged >12 years and had relatively long disease duration; however, the result was not significant, probably because of the small sample size and the selected conditions [24]. *H. pylori* infection was found to have a substantial negative impact on metabolic control in children and adolescents with T1DM [26,35]. Dai et al. reported that *H. pylori* infection was positively correlated with HbA1c levels and worse glycemic control in adolescents and children with T1DM [36]. These findings indicated a notable link between *H. pylori* infection and increased levels of HbA1c, as well as the duration of T1DM.

A meta-analysis by Feng Wang, who included 39 eligible studies from 1997 to 2012, showed a significant association between *H. pylori* infection and an increased risk of both T1DM and T2DM [37]. In another meta-analysis by Kamyar Mansori on 41 studies with 9559 individuals from 1990 to 2019, there was a significant association between *H. pylori* infection and the risk of developing diabetes, particularly T2DM, in the subgroup analysis [38]. Similarly, this present meta-analysis found a positive association between T1DM and *H. pylori* infection, although it did not reach statistical significance. Another study revealed that *H. pylori* infection was not significantly associated with T1DM in children and that the glycemic control in these patients was similar between those who developed *H. pylori* infection and those who did not [34]. The potential association between *H. pylori* infection and T1DM remains a topic of debate, particularly in terms of glycemic control, gastrointestinal symptoms, infection prevalence, eradication and reinfection rates, and sanitary conditions [29]. In this meta-analysis, we performed an extensive and up-to-date literature search to identify a significant number of studies that provided sufficient data from 2797 pediatric individuals from 1997 to 2020 and found a positive correlation of *H. pylori* infection with HbA1c levels and T1DM duration. Indeed, this meta-analysis had a relatively large sample size, and none of the included studies had an individual substantial influence on the overall results. 

Based on our findings, an alternative inference was the impact of DM on the incidence of *H. pylori* infection. Therefore, this infection might be a complication rather than a cause of T1DM. This result could be attributed to the fact that in individuals with diabetes, reduced gastric motility and peristaltic activity might facilitate *H. pylori* colonization [39]. Infection with *H. pylori* increases the production of proinflammatory cytokines, which may lead to impaired glycemic control [40]. Furthermore, chemical changes, such as the nonenzymatic glycosylation of mucins, in the gastric mucosa and elevated sialic acid levels may act as receptors on cell surfaces and facilitate the adhesion of *H. pylori* to gastric mucosa cells [41,42,43]. In addition, impaired nonspecific immunity further contributes to the risk of *H. pylori* infection in patients with diabetes [41].

Several mechanisms that can explain the association between *H. pylori* infection and the risk of diabetes have been proposed. One mechanism involves inflammatory cytokines, which can induce the phosphorylation of serine residues on the insulin receptor substrate. This phosphorylation may impair the interaction between the substrate and the insulin receptors, leading to impaired insulin function [44]. Furthermore, *H. pylori* infection induces inflammation, which affects the pancreatic β cells and leads to decreased insulin secretion. In particular, cag+ strains of *H. pylori* can further reduce insulin secretion by affecting the production of somatostatin [38,45]. Another mechanism involves lipopolysaccharides (LPSs), which are produced by Gram-negative bacteria, such as *H. pylori*. LPSs can activate Toll-like receptors, resulting in insulin resistance [46]. Moreover, *H. pylori* infection has been associated with elevated leptin and ghrelin levels, which can contribute to obesity and increase the risk of developing diabetes [47].

These events collectively contribute to poor blood sugar control during the development of diabetes mellitus. In this study, *H. pylori* infection was not significantly associated with age at T1DM diagnosis, but it had a noteworthy correlation with prolonged T1DM duration and HbA1c level. These findings imply that although *H. pylori* might not affect the age of initial T1DM diagnosis, it could influence the progression of T1DM. It is plausible that over time, impaired glucose metabolism in patients with T1DM could facilitate *H. pylori* colonization, suggesting that a vicious cycle of *H. pylori* infection and impaired glucose control facilitates T1DM development. Nevertheless, further research is essential to fully comprehend the underlying mechanisms.

The studies included in this meta-analysis used a variety of diagnostic methods (e.g., the urea breath test; measurement of IgA, IgG, and IgM antibodies for *H. pylori*; the detection of *H. pylori* antigens in stool samples; and the measurement of cagA IgG antibodies) for *H. pylori* infection. This might have contributed to the divergent findings on this topic. Of the 12 case–control studies, 8 used an enzyme-linked immunosorbent assay to detect *H. pylori* antibodies. Notably, serologic methods cannot differentiate between recent and previous infections. In one study, detection of fecal *H. pylori* antigens was found to potentially offer greater relevance in identifying active gastrointestinal infection in specific patients with diabetes [48]. Therefore, future studies should include multiple tests to provide more comprehensive and reliable results. 

Clinically, the ESPGHAN/NASPGHAN and JSPGHAN guidelines for the management of *H. pylori* in children and adolescents recommend against treating *H. pylori* without peptic ulcer disease, gastric mucosa-associated lymphoid tissue lymphoma, or chronic idiopathic thrombocytopenia purpura. However, we found that *H. pylori* was associated with relatively high HbA1c levels and long duration of diabetes. Therefore, we recommend that *H. pylori* infection should be considered in pediatric cases of T1DM with poor blood sugar control. We hypothesized that *H. pylori* may exert this influence through a toxin-associated mechanism, because it is known to produce some toxins secondary to inflammation, cytokine reaction, and autoimmune disease.

The current study had several limitations. First, there was no information on the history of drug or nondrug treatments, which can affect the presence of *H. pylori* infection and the development of metabolic syndrome and insulin resistance. Future studies should consider evaluating the impact of different treatments on these outcomes in individuals with *H. pylori* infection. Second, gastrointestinal comorbidities, such as celiac disease, were not considered; these should also be addressed in future studies. Third, the current study focused on patients with T1DM and did not compare the association between *H. pylori* infection and HbA1c levels between patients with T1DM and those with T2DM. Therefore, future studies should include patients with T2DM to facilitate a more comprehensive analysis and provide valuable insights on the different types of diabetes. Fourth, our study did not have access to a dataset from Asian populations, thereby limiting the generalizability of our findings to this group; future research should include datasets from Asia. Fifth, although case–control studies can provide valuable insights on associations, they have inherent limitations, such as recall and selection biases. Therefore, the design and implementation of cohort studies could yield a more comprehensive and detailed assessment of the association between *H. pylori* infection and diabetes. 

## 5. Conclusions

Notably, the subgroup analysis found a significant and positive link of *H. pylori* infection with HbA1c level and diabetes duration. These results imply that chronic conditions that result in disrupted glycemic control and prolonged disease duration have a potential influence on the susceptibility to *H. pylori* infection.

For a more comprehensive understanding and to obtain robust evidence, the next step would entail large-scale cohort studies with substantial sample sizes; rigorous methodologies that take into account factors such as weight loss, socioeconomic status, and household size; and the inclusion of worldwide populations. Such studies could yield more explicit insights and enhance our comprehension of the complex interplay between *H. pylori* infection and diabetes outcomes, thereby paving the way for more targeted interventions and improved management strategies for both *H. pylori* infection and diabetes-related complications. Nevertheless, the findings of this meta-analysis suggest that pediatric patients with relatively long T1DM duration and poor glycemic control should be screened for *H. pylori* infection.

## Figures and Tables

**Table 1:** The main charateristics of included studies and the relation between *H. pylori* and Diabetes.

Author	Year	Nation	Study Design	Sample Size	Gender (Male/Female)	Age	Control Group	Measurement of Association Odds Ratio (95% C.I.)	Type of Diabetes (Mean of HbA1c) (Duration)	*H. pylori* Test Method	Control Var.
M Pocecco, et al. [23]	1997	Italy	Case-Control Study	379 (Control: 310; Case: 69)	213/166	16	Admitted for minor extra-abdominal surgery with no history of abdominal pain	6.08 (2.94, 12.58)	DM (-) (-)	ELISA	Age, sex, education and economic status
S Salardi, et al. [24]	1999	Italy	Case-Control Study	339 (Control: 236; Case: 103)	N/A	12	Patients attending the hospital for minor endocrine disorders	1.79 ( 0.93, 3.44)	T1DM (-) (4.96 ± 3.22 years)	RIBASIA	Age
Arslan D., et al. [25]	2000	Turkey	Case-Control Study	130 (Control: 42; Case: 88)	N/A	12	Healthy children	2.80 (1.29 to 6.10)	T1DM (11.08 ± 3.17) (3.85 Years)	ELISA	-
Marcello Candelli, et al. [26]	2003	Italy	Case-Control Study	268 (Control: 147; Case: 121)	145/123	14.96	Healthy participants	0.98 (0.58, 1.66)	T1DM (8.2 ± 1.4 ) (79.7 ± 55.5 months)	C-UBT	Age, sex, and social class
Krause I, et al. [27]	2009	Colombia	Case-Control Study	197 (Control: 140; Case: 57)	N/A	16	Healthy subjects	3.35 (1.72, 6.53)	T1DM (-) (8.8 ± 8.7 years)	ELISA	-
Cabral VL, et al. [28]	2009	Brazil	Case-Control Study	45 (Control: 30; Case: 15)	N/A	17.6	Adolescents with the histological findings of gastric and duodenal biopsies with normal mucosal architecture	0.38 (0.10, 1.39)	T1DM (-) (8 ± 3.6 years)	Rapid Urease Test	-
El-Eshmawy M.M., et al. [29]	2011	Egypt	Case-Control Study	242 (Control: 80; Case: 162)	108/134	19.49	Healthy subjects	3.58 (2.01, 6.39)	T1DM (8.2 ± 1.75) (7.29 ± 7.9 years)	ELISA	Age, sex and socioeconomic status
Zekry O.A., et al. [30]	2013	Egypt	Case-Control Study	120 (Control: 60; Case: 60)	N/A	12.53	Healthy children who were selected from among relatives	2.4 (1.25,4.58)	T1DM (-) (-)	ELISA	Age, sex and socioeconomic
Agata Chobot, et al. [31]	2014	Poland	Case-Control Study	447 (Control: 298; Case: 149)	201/246	13.4	Healthy children and adolescents	0.65 (0.36, 1.18)	T1DM (7.69 ± 1.63 ) (4.6 ± 3.5 years)	C-UBT	Age and sex
Samah M Osman, et al. [32]	2016	Sudan	Case-Control Study	180 (Control: 90; Case: 90)	96/84	1-18	Healthy children	0.95 (0.51, 1.76)	T1DM (-) (duration < 6 month)	ELISA	Age and sex
Hassan Bazmamoun, et al. [33]	2016	Iran	Case-Control Study	160 (Control: 80; Case: 80)	63/97	9.37	Non-Diabetic children from the same clinic	2.25 (1.20 to 4.24)	T1DM (-) (2.14 ± 0.43)	EIA Test	Age, sex and socioeconomic status
Esmaeili Dooki MR, et al. [34]	2020	Iran	Case-Control Study	168 (Control: 105 ; Case: 63)	81/87	10.44	Children without Diabetes Mellitus	1.18 (0.58, 2.42)	T1DM (-) (at least 6 months)	Stool Test	Age and gender

N/A: Not available.

**Table 2:** Characteristic of Diabetic patients.

Author	Year	Nation	Study Design	Sample Size	Gender (Male/Female)	Diabetic-Age (Year)		Diabetic-HbA1c (%)		Diabetic-Duration (Year)	
						HP+	HP−	HP+	HP−	HP+	HP−
M Pocecco, et al. [23]	1997	Italy	Case-Control Study	69	42/27	16	11	7.6	7.1	3	2
S Salardi, et al. [24]	1999	Italy	Case-Control Study	103	N/A	13.2 ± 3.4	11.2 ± 3.4	N/A	N/A	6 ± 3.4	4.3 ± 3.2
Arslan D., et al. [25]	2000	Turkey	Case-Control Study	88	N/A	N/A	N/A	N/A	N/A	N/A	N/A
Marcello Candelli, et al. [26]	2003	Italy	Case-Control Study	121	65/56	16 ± 5.6	14.3 ± 5.5	8.05 ± 4.52	7.9 ± 10	8.05 ± 4.52	5.35 ± 4.09
Krause I, et al. [27]	2009	Colombia	Case-Control Study	57	24/33	N/A	N/A	N/A	N/A	N/A	N/A
Cabral VL, et al. [28]	2009	Brazil	Case-Control Study	15	6/9	18	17	N/A	N/A	7	10
El-Eshmawy M.M., et al. [29]	2011	Egypt	Case-Control Study	162	72/90	20.1 ± 4.6	19.8 ± 4.34	8.3 ± 1.58	6.8 ± 2.3	8.9 ± 8.6	4.22 ± 2.35
Zekry O.A., et al. [30]	2013	Egypt	Case-Control Study	60	N/A	12.0 ± 2.4	12.89 ±2.29	7.75 ± 1.67	5.72 ±1.2	9.25 ± 2.73	6.11 ± 1.78
Agata Chobot, et al. [31]	2014	Poland	Case-Control Study	149	67/82	13.3 ± 3.3	13.9 ±3.6	7.82 ± 1.42	7.60 ± 1.66	5.3 ± 3.9	4.4 ± 3.4
Samah M Osman, et al. [32]	2016	Sudan	Case-Control Study	90	50/40	N/A	N/A	N/A	N/A	N/A	N/A
Hassan Bazmamoun, et al. [33]	2016	Iran	Case-Control Study	80	32/48	7.7 ± 0.86	7.58 ± 0.65	8 ± 0.65	7.9 ± 0.40	2.72 ± 0.55	1.26 ± 0.13
Esmaeili Dooki MR, et al. [34]	2020	Iran	Case-Control Study	63	34/29	8.84 ± 2.03	7.45± 2.9	8.08 ± 1.51	7.9 ± 0.40	2.74 ± 1.62	3.16 ± 2.57

N/A: Not available.

## Data Availability

The data of this study are available on request from the corresponding authors, H.C.F. and W.H.T.

## Supplementary Materials

The following supporting information can be downloaded at: https://www.mdpi.com/article/10.3390/medicina60010119/s1, Figure S1: Correlation analysis of *H. pylori* infection and age at diagnosis of T1DM.; Figure S2: (A) Sensitivity analysis for the study; (B) Sensitivity analysis for Age; (C) Sensitivity analysis for duration of diabetes; (D) Sensitivity analysis for HbA1c level.
