# Supplementary figures and images for: A Significant Association between Type 1 Diabetes and Helicobacter pylori Infection: A Meta-Analysis Study
Source: Medicina (Kaunas). 2024 Jan 9;60(1):119. doi: 10.3390/medicina60010119 (PMC10821400; doi:10.3390/medicina60010119)

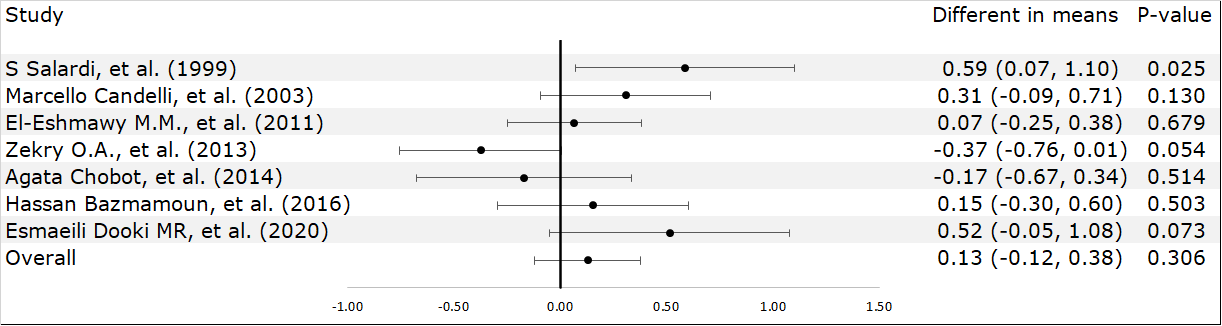

Supplement: Supplementary file 1 [file medicina-60-00119-s001.zip › Figure S1.png]

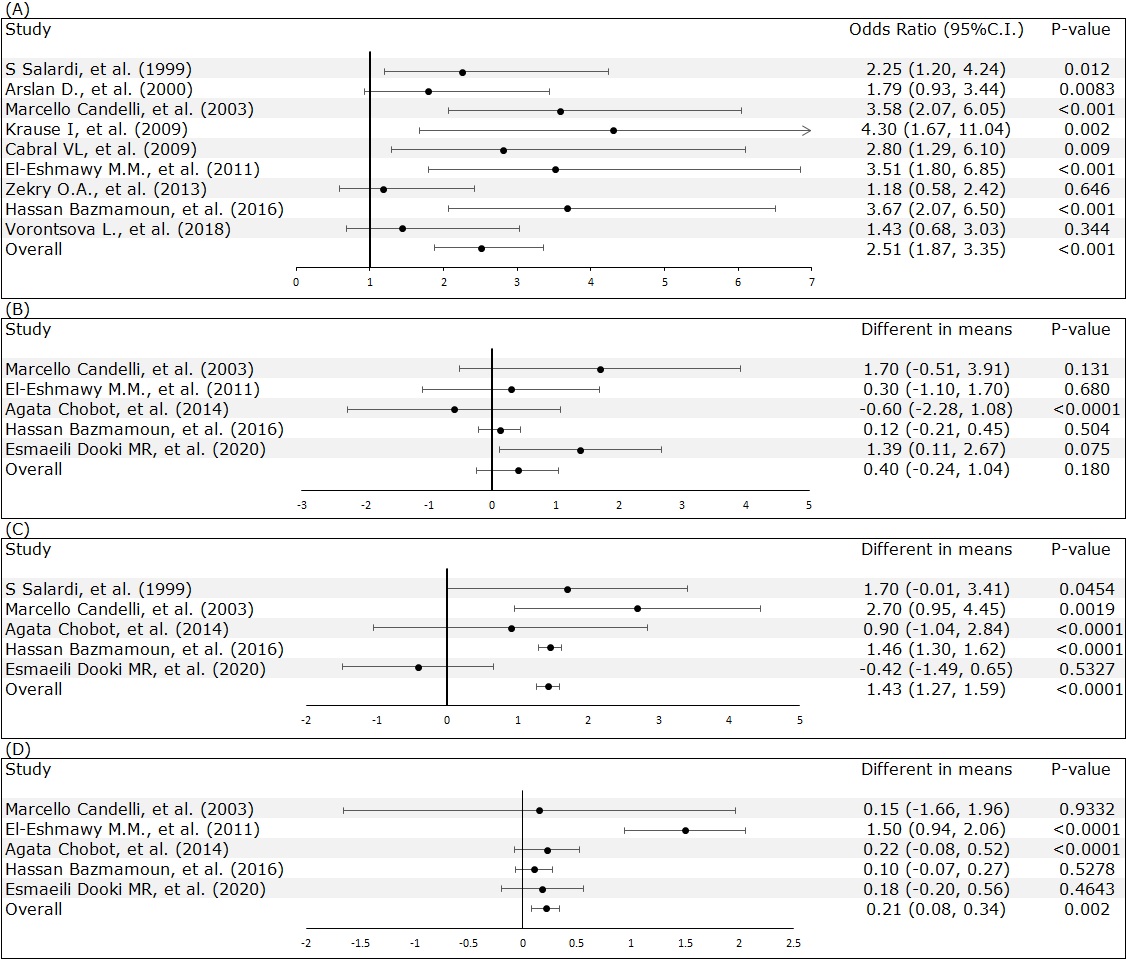

Supplement: Supplementary file 1 [file medicina-60-00119-s001.zip › Figure S2.jpg]
